# Ultrasonographic identification of the cricothyroid membrane in a patient with a difficult airway as a result of cervical hematoma caused by hemophilia: a case report

**DOI:** 10.1186/s12871-019-0798-3

**Published:** 2019-07-09

**Authors:** Ippei Jimbo, Kohji Uzawa, Joho Tokumine, Shingo Mitsuda, Kunitaro Watanabe, Tomoko Yorozu

**Affiliations:** Department of Anesthesiology , Kyorin University, School of Medicine 6-20-2 Shinkawa, Mitaka City, Tokyo, 181-0004 Japan

**Keywords:** Cricothyroid membrane, Cricothyroidotomy, Ultrasonography, Difficult airway, Hemophilia

## Abstract

**Background:**

Surgical cricothyroidotomy is a last resort in patients with an anticipated difficult airway, but without any guarantee of success. Identification of the cricothyroid membrane may be the key to successful cricothyrotomy. Ultrasonographic identification of the cricothyroid membrane has been reported to be more useful than the conventional palpation technique. However, ultrasonographic identification techniques are not yet fully characterized.

**Case presentation:**

A 28-year-old man with hemophilia and poor adherence to medication. He was brought to the emergency department with a large cervical hematoma and respiratory difficulty. An otolaryngologist decided to insert a tracheal tube to maintain his airway. However, emergent laryngoscopy indicated an anticipated difficult airway. A backup plan that included awake intubation by the anesthesiologists and surgical cricothyroidotomy by an otolaryngologist was devised. The cricothyroid membrane could not be identified by palpation but was detected by ultrasonographic identification with a longitudinal approach. Awake fiberoptic intubation was successfully performed.

**Conclusions:**

In this case, the cricothyroid membrane could be identified using the longitudinal approach but not the transverse approach. It may be ideal to know which ultrasound technique can be applied for each patient.

## Background

Management of worsening respiratory distress in a patient with a difficult airway is problematic. When a difficult airway is anticipated, the decision regarding whether or not to perform awake intubation or surgical cricothyroidotomy is challenging. Furthermore, there is no guarantee of success using either procedure and there is an ever-present possibility of “cannot intubate cannot oxygenate” (CICO) [1]. Guidelines for the management of a difficult airway, as followed in the US [2], UK [3], Canada [1], and Japan [4], recommend securing of the airway by surgical incision or puncture of the cricothyroid membrane (CTM) as a last resort in a CICO situation. Preparation for both less invasive awake fiberoptic intubation and invasive cricothyroidotomy is known as the “double standby” strategy [1]. However, an anesthesiologist is unlikely to be able to palpate the cricothyroid membrane accurately [5]. Misidentification of the cricothyroid membrane is a major reason for tube misplacement resulting in failed cricothyroidotomy, and in a CICO situation, serious complications, such as tension pneumothorax and pneumomediastinum [6, 7]. We have encountered a patient in whom a difficult airway was anticipated as a result of cervical hematoma caused by untreated hemophilia in whom we elected to use the double standby method. The CTM could not be identified by the conventional palpation technique but could be identified on ultrasonographic examination.

## Case presentation

**Fig. 1:**
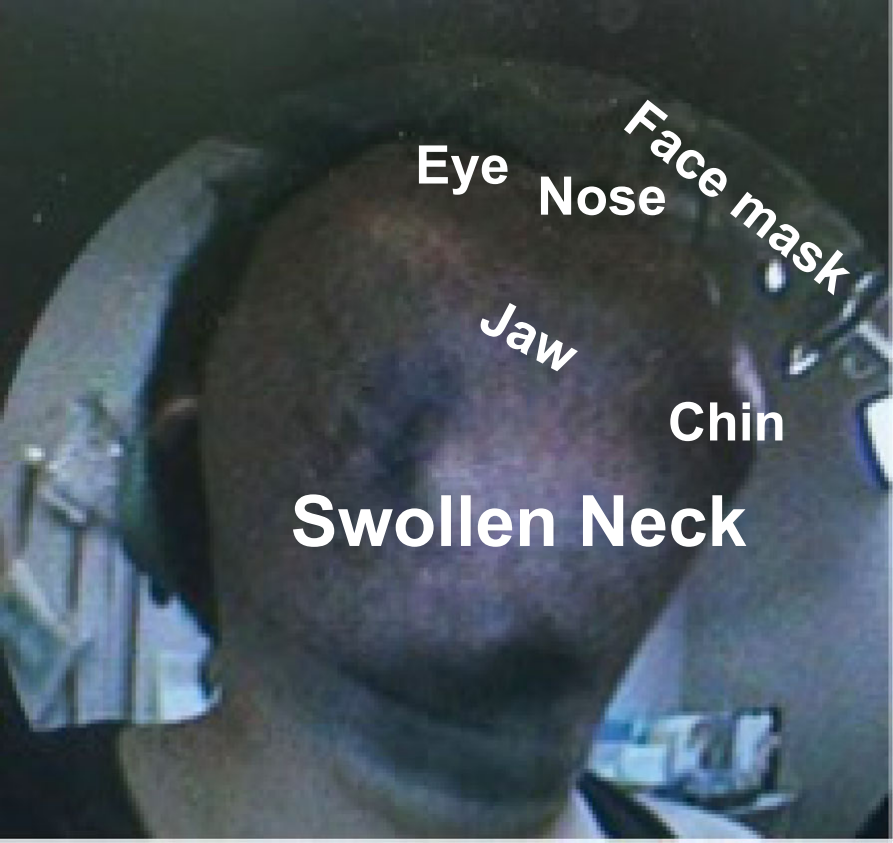
Clinical photograph showing the neck swelling caused by the large hematoma

A 28-year-old man (height 165 cm, body weight 80 kg, body mass index 29) with congenital hemophilia A was admitted to hospital with cervical swelling, difficulty vocalizing, and stridor during inspiration (Fig. 1). The patient had complied poorly with medication and discontinued treatment 6 months earlier. Laboratory tests revealed impaired coagulation (activated partial thromboplastin time 95.8 s, prothrombin time-international normalized ratio 1.04).

**Fig. 2:**
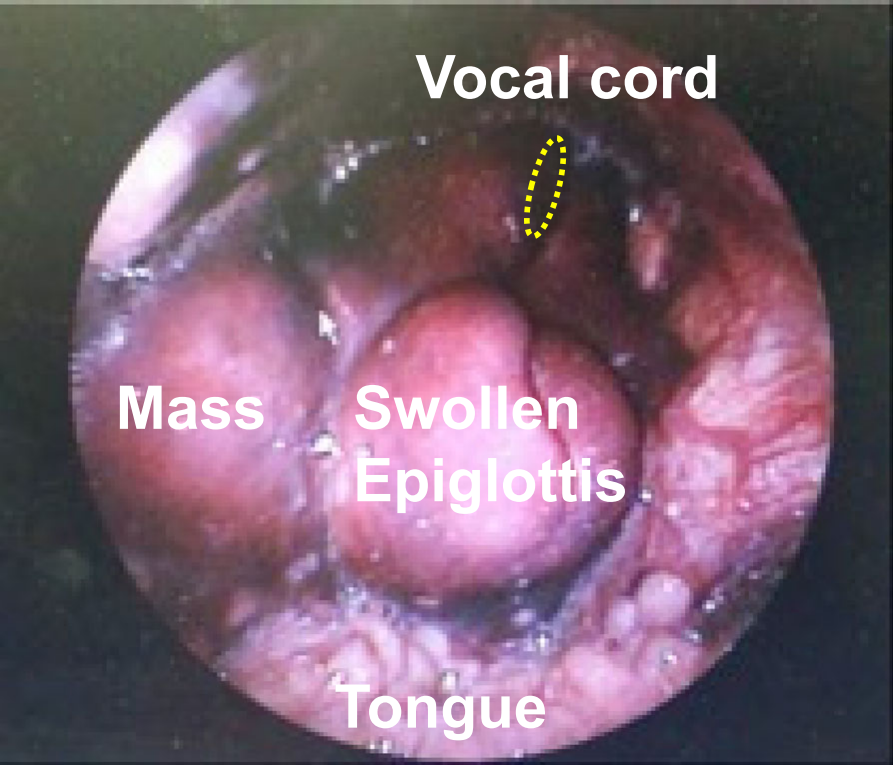
Transnasal endoscopic findings in the laryngeal cavity. The image shows a swollen epiglottis with a mass on the right side of the epiglottis. The vocal cords can be seen under the swollen epiglottis

The patient’s SpO_2_ was 97% in room air and his respiratory rate was 10 breaths/min. One hour later, his respiratory distress had progressed to orthopnea and his SpO_2_ had decreased to 92%. An otolaryngologist performed a transnasal endoscopic examination that revealed severe swelling and a large mass around the vocal cords (Fig. 2). These findings suggested the possibility of imminent suffocation and a need for urgent securing of the airway.

**Fig. 3:**
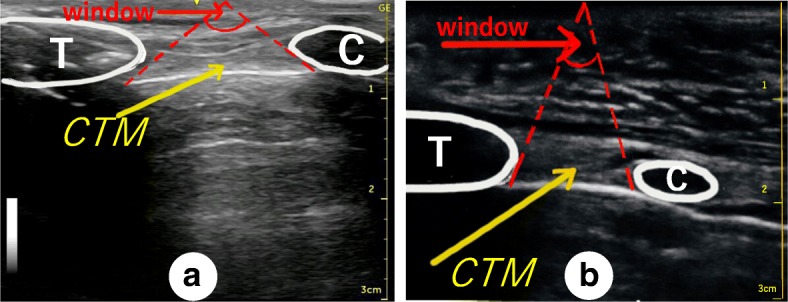
Cervical ultrasonographic images obtained using the longitudinal approach. Either the transverse or longitudinal approach can be used for ultrasonographic identification of the cricothyroid membrane (CTM). Using the transverse approach, the operator manipulates the ultrasound probe while tilting it up and down on the patient’s neck to locate the CTM; if the operator is attempting to locate a deeply positioned CTM (a), the angle of tilt of the probe may be restricted, and is shown as a window (a, red lettering). In contrast, if the CTM is in a shallow position (b), the angle of tilt of the probe may be wider using the transverse approach. However, there is no need to tilt the probe when using the longitudinal approach. **a** An ultrasonographic image of the patient’s neck using the longitudinal approach. **b** An ultrasonographic image of the first author’s neck using the longitudinal approach. The first author is a healthy male adult with a standard physique (height 174 cm, body weight 68 kg). T, thyroid cartilage; C, cricoid cartilage; CTM, cricothyroid membrane

The attending otolaryngologist and anesthesiologists discussed how to secure the airway and agreed to prepare for double standby. However, the otolaryngologist could not identify the CTM by conventional palpation, so an anesthesiologist searched for the CTM using ultrasonographic examination. The search was started from just above the jugular notch of the sternal manubrium because this site looked anatomically normal. However, although the thyroid cartilage could be identified easily on a transverse view, no clear picture of the CTM could be obtained. The anesthesiologist subsequently identified the CTM on a longitudinal view (Fig. 3) and marked its location for surgical cricothyroidotomy.

Awake fiberoptic intubation was successfully performed via an oral approach under topical anesthesia with 8% lidocaine spray and intravenous administration of fentanyl 100 μg. The patient’s hypoxia did not worsen during the procedure. The patient was treated with steroid replacement therapy and coagulation factor VIII, and his glottic edema gradually resolved. The patient was extubated on day 6 and discharged without complications on day 13.

## Discussion and conclusion

The guidelines for management of a difficult airway recommend incision or puncture of the CTM in a CICO situation. However, the fourth National Audit Project [8] reported that surgical securing of the airway under anesthesia in such circumstances had a 43% risk of serious complications.

Cricothyroidotomy by incision of the CTM is more reliable than puncture, and its success depends on correct identification of the CTM [9]. However, the CTM may be difficult to identify by the conventional palpation technique if it is not in the normal anatomical location [10]. In the present case, the otolaryngologist could not identify the superior thyroid notch by palpation because of the overlying hematoma. The conventional palpation technique is usually started from the superior thyroid notch as an anatomical landmark. Ultrasonographic guidance may have an advantage over conventional palpation for identifying the CTM in the event of an anatomical abnormality [10].

In our case, the CTM could not be identified by the widely used transverse ultrasound approach [5] because the patient’s hematoma extended from the mandible to the upper neck. The superior thyroid notch could not be identified by palpation, so the anesthesiologist started to scan from the patient’s lower neck. A longitudinal ultrasound approach has been described but its efficacy is thought to be limited because the ultrasound probe cannot be positioned correctly on the skin surface in a patient with a short neck or severe cervical flexion deformity [5]. Kristensen et al. reported that the transverse and longitudinal approaches for ultrasonographic identification of the CTM in obese female subjects had a 90% success rate for identifying the CTM [7]. Interestingly, they found that neither approach was inferior to the other for identification of the CTM in obese patients [7]. The anesthesiologists’ first choice in the report by Kristensen et al. was a transverse approach because they were familiar with it and unfamiliar with the longitudinal approach. However, our anesthesiologists could identify the CTM using the longitudinal approach but not the transverse approach. In our patient, the CTM was deep below the skin surface, which made it difficult to locate using the transverse approach because of the narrow searching space between the thyroid cartilage and the cricoid cartilage (Fig. 3).

Siddiqui et al. reported that ultrasonography successfully identified the CTM even in cadavers with poorly defined neck anatomy and speculated that ultrasonographic identification may reduce complications and improve the success rate of cricothyroidotomy [11]. The 2015 UK Difficult Airway Society guidelines recommend preoperative use of ultrasonography for identification of the CTM to ensure successful cricothyroidotomy in patients anticipated to have a difficult airway [3] but not otherwise, and with the caveat that ultrasonographic identification of the CTM might be unnecessarily time-consuming when the airway needs to be surgically secured in an emergency. We believe that ultrasonographic identification of the CTM is not time-consuming when performed by a skilled operator. It has been reported that competence can be achieved by a short period of hands-on training [7, 12]. Therefore, training should make reliable identification of the CTM easier and ensure successful cricothyroidotomy.

The CTM could not be found by palpation in our patient but could be identified by ultrasonography. Fortunately, awake intubation was successful in this case. However, we cannot state with certainty that cricothyroidotomy would be successful using the method described here in a patient approaching CICO after awake intubation has failed. It is difficult to conduct high-quality clinical research on the success rate of cricothyroidotomy under ultrasonographic guidance, so the efficacy of identification of the CMT using this modality is still a matter of debate. However, it was recently found that ultrasound-guided identification of the cricothyroid membrane [6, 13] is highly effective and is comparable to a CT-scan as the accepted standard [14] in patients with abnormal neck anatomy. This strongly indicates that this technique can be applied for patients such as the one described in this report.

## Data Availability

All data generated or analyzed during this study are included in this published article (and its supplementary information files). The datasets are available from the corresponding author on reasonable request.

## Abbreviations

CICO: “cannot intubate cannot oxygenate”
CTM: cricothyroid membrane
